# County of San Diego, California, investigates *Salmonella Typhimurium* outbreak linked to unpasteurized milk – September 2023–January 2024

**DOI:** 10.1017/S0950268826101009

**Published:** 2026-04-13

**Authors:** Audrey E. Kennar, Azarnoush Maroufi, Madeline Poranski, Heather Stachelrodt Watson, Phoebe Seaver, Meghan Villalobos, Ernie Awa, Paul Temprendola, Vince Huynh, Nicholas S. Rhoades, Heather Buonomo, Ryan Johnson, Mark E. Beatty, Anna Liza Manlutac, Jeremy Corrigan, Annie Kao, Sarah Stous, Seema Shah

**Affiliations:** 1 Centers for Disease Control and Prevention, Atlanta, GA, USA; 2 County of San Diego Health and Human Services Agency, San Diego, CA, USA; 3 County of San Diego Department of Environmental Health and Quality, San Diego, CA, USA

**Keywords:** California, dairy products, product recalls, *Salmonella (Typhimurium)*, whole genome sequencing

## Abstract

On 17 October 2023, the County of San Diego (CoSD), California’s Epidemiology and Immunization Services Branch received reports of eight salmonellosis patients with illness onsets beginning in September 2023. CoSD disease investigators identified common exposure to Farm A-produced unpasteurized dairy products and performed epidemiologic and environmental investigations. Isolates were submitted for whole-genome sequencing. Cases were defined as persons with *Salmonella* infection with an isolation date on or after 15 September 2023. Twenty-five cases, including 23 confirmed and two probable, were identified among county residents, with 19 (76%) reporting exposure to Farm A unpasteurized dairy products. Median patient age was 12 years (range: 1–47 years). Three children (12%) were hospitalized. Environmental samples of unpasteurized milk collected from Farm A tested positive for *S. Typhimurium*, matching the outbreak strain by WGS. After *Salmonella* identification, CoSD released early advisories warning of Farm A-associated risks, likely preventing additional infections.

## Introduction

*Salmonella* bacteria cause approximately 1.35 million infections annually in the United States [1]. Most of these infections are caused by foodborne transmission. Common symptoms of salmonellosis include bloody or watery diarrhea, fever, stomach cramps, nausea, vomiting, and headache, and usually start from 6 h to 6 days after consuming contaminated food. Salmonellosis symptom duration typically lasts 4–7 days after onset. In certain instances, a more severe illness requiring hospitalization develops. Persons at an increased risk for *Salmonella* infection and severe illness are children aged <5 years, adults aged ≥65 years, and persons with a weakened immune system [1–3].

Unpasteurized (also known as raw) dairy products have not undergone the pasteurization process; therefore, these dairy products are not heated to a temperature point for a period sufficient to kill harmful bacteria. Harmful bacteria found in unpasteurized dairy products can lead to preventable foodborne illnesses and disease outbreaks, including *Salmonella* spp., *Campylobacter* spp., *Escherichia coli* (*E. coli*), *Listeria monocytogenes*, and other bacteria [4, 5]. During 2013–2018, 75 disease outbreaks were linked to unpasteurized milk in the United States, resulting in 675 illnesses and 98 hospitalizations [6].

In California, unpasteurized dairy products can be sold if the producing dairy farm meets sanitation and licensing requirements outlined by the California Department of Food and Agriculture (CDFA). All unpasteurized dairy products must include a warning label to alert consumers to the risk of exposure to harmful bacteria [7, 8]. Unpasteurized dairy products are required to meet the same bacteria count limits as pasteurized milk, with CDFA’s Milk and Dairy Food Safety Branch performing monthly pathogen testing [8].

**Figure 1.**
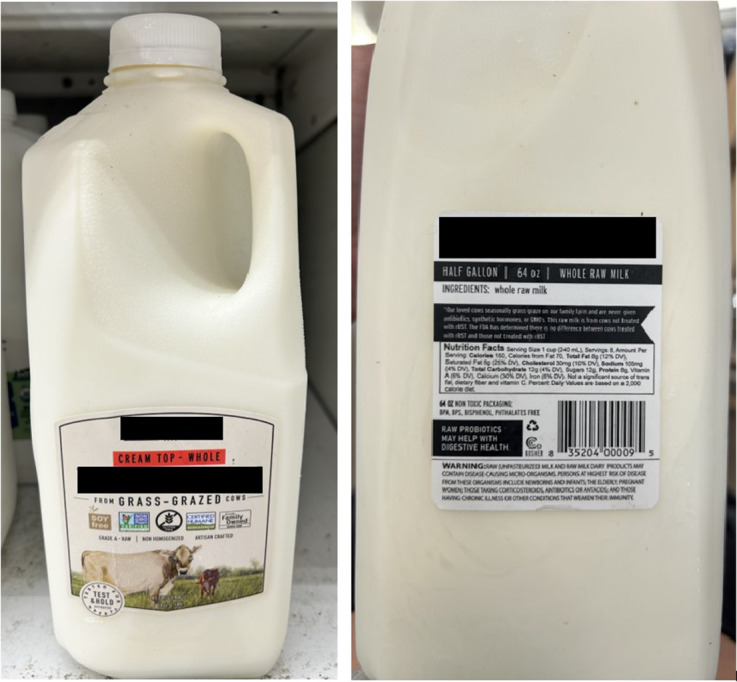
Front and back of implicated products [9]. Figure 1. long description.

On 17 October 2023, County of San Diego’s (CoSD) Epidemiology and Immunization Services Branch (EISB) identified eight cases of salmonellosis (six culture-confirmed and two polymerase chain reaction (PCR)-positive) among San Diego County residents. Illness onset dates were from late September to early October 2023. Among the eight patients, three were hospitalized, all of whom were children. EISB’s Enterics Team Communicable Disease Investigators used standardized case interviews and determined that all eight patients reported consumption of Farm A brand unpasteurized milk or milk products purchased through commercial grocery vendors during the week before symptom onset (Figure 1). The vendor distribution list included chain retailers. To determine the implicated unpasteurized dairy products produced by Farm A, EISB began an investigation.

## Methods

### Epidemiologic and laboratory investigation

**Figure 2.**
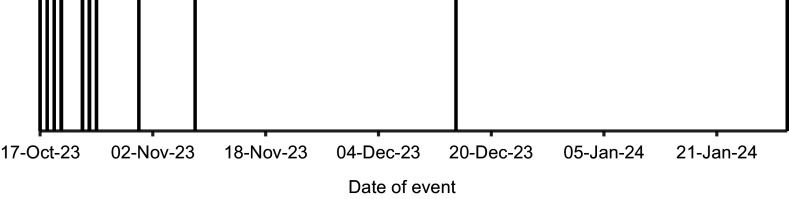
Timeline of events. 17 October 2023: CoSD EISB foodborne outbreak team was notified of the common exposure among eight salmonellosis patients. 18 October 2023: EISB opened an outbreak investigation after identifying common exposure to Farm A unpasteurized dairy products. 19 October 2023: Grocery vendors were alerted and asked to voluntarily pull and hold Farm A unpasteurized dairy products. The Raw Milk Information Collection Tool was used during re-interviews. Unopened Farm A products were collected from vendors. 20 October 2023: CoSD released a health alert and press release. 23 October 2023: Serotype *S. Typhimurium* was identified in the first confirmed *Salmonella* isolate from an infected patient. This outbreak was caused by a strain related by WGS with zero to one allele codes. 24 October 2023: Farm A agreed to a voluntary recall of raw milk and raw heavy cream. 25 October 2024: CoSD issued an updated press release. 31 October 2023: Farm A production resumed. 8 November 2023: CoSD had identified 19 infected persons, 17 confirmed and two probable. 15 December 2023: Case definitions were updated to include WGS results. 31 January 2024: Twenty-five total cases (23 confirmed and two probable cases) from San Diego County were identified. Prepared by CoSD Health and Human Services Agency. CoSD, County of San Diego; EISB, Epidemiology and Immunization Services Branch.

Because the threshold of common exposure in sick persons was met (i.e., ≥two people from different households reporting illness with the same food item consumption during the same two-week exposure period), EISB opened a foodborne outbreak investigation on 18 October 2023 (Figure 2). The common exposure to Farm A products also prompted notification to local and state partners, including the CoSD EISB Medical Director and Epidemiology Unit, CoSD Department of Environmental Health and Quality (DEHQ), California Department of Public Health (CDPH), CDFA, and other local health jurisdictions. This same day, an additional infected patient was identified by retrospective review of salmonellosis case reports for unpasteurized dairy product exposure. This brought the total to nine salmonellosis cases among San Diego County residents.

EISB began using CDPH’s Raw Milk Information Collection Tool on 19 October to reinterview patients who reported consuming unpasteurized dairy products, or caregivers, to obtain demographic information and identify specific products. This tool is a supplement to the salmonellosis interview report form and includes questions specific to the 1-week period before the patient’s symptom onset. It included questions about the consumption of unpasteurized dairy products and whether leftover product was available for testing. The collected information was entered into the Web Confidential Morbidity Report, CoSD’s disease registry. Purchase receipts for date, vendor, and product verification were requested, and opened and unopened products remaining in patient and caregiver households were collected for testing.

On 20 October, CoSD released a health alert and press release asking persons with gastrointestinal symptoms and exposure to unpasteurized dairy products from Farm A products to seek medical care and get tested for *Salmonella.* The media release advised persons to discard any recently purchased Farm A unpasteurized dairy products [10, 11]. Confirmed cases were initially defined as a person having Farm A unpasteurized dairy product exposure and culture-confirmed *Salmonella*; probable cases were defined as persons positive for *Salmonella* on PCR testing with Farm A unpasteurized dairy product exposure. As the investigation continued, EISB’s investigators identified additional infected patients through routine enteric surveillance. Culture-confirmed *Salmonella* isolates from previously collected stool specimens were submitted to Los Angeles County Public Health (LACPH) or CDPH Microbial Diseases Laboratory (MDL) for serotyping and whole-genome sequencing (WGS).

Although initial case finding looked for unpasteurized dairy exposure, as the investigation progressed, the case definitions were updated on 15 December 2023, to include infections matched by WGS results. Cases were limited to persons with *Salmonella* infection with an isolation date on or after 15 September 2023. The confirmed case definition was updated to include core-genome multilocus sequence typing [cgMLST] or six single-nucleotide polymorphisms [SNPs] using National Center for Biotechnology Information [NCBI] SNP analysis. A confirmed case was defined as a person with *S. Typhimurium* with a common allele code that was highly related (within two cgMLST alleles). A probable case was updated to include a person who consumed Farm A unpasteurized milk products and had a PCR-positive result without available *Salmonella* serotype or sequence results. While the probable cases did not have isolates available for WGS testing, these cases were counted as part of the outbreak by epi-linkage.

### Environmental investigation

Concurrent with the epidemiologic investigation, on 19 October, grocery vendors were alerted to the ongoing outbreak. CDPH provided a distribution list to CoSD DEHQ, who then contacted the grocery vendors identified as having received Farm A unpasteurized dairy products and asked the vendors to voluntarily remove from sale and hold these products. Additionally, CoSD DEHQ collected unopened Farm A unpasteurized dairy products from multiple vendors. The implicated product from one infected patient was also collected. All collected products were submitted to the CDPH Food and Drug Branch (FDB) for *Salmonella* testing and sequencing. CDFA also collected samples of unpasteurized milk from the Farm A facility in California for testing and sequencing.

### Ethics

This activity was reviewed by CDC, deemed not research, and was conducted consistent with applicable federal law and CDC policy. (See e.g., 45 C.F.R. part 46.102(l)(2), 21 C.F.R. part 56; 42 U.S.C. §241(d); 5 U.S.C. §552a; 44 U.S.C. §3501 et seq.)

## Results

### Epidemiologic and laboratory results

By 20 October, EISB’s investigators identified three additional infected patients who reported consumption of Farm A unpasteurized dairy products, bringing the total to 12 infected patients.

On 23 October, serotype *Salmonella Typhimurium* was identified in the first culture-confirmed *Salmonella* isolate from an infected patient. This outbreak was caused by a strain related by WGS within zero to one cgMLST alleles. By 8 November, CoSD had identified 19 infected persons, 17 confirmed and 2 probable cases.

**Figure 3.**
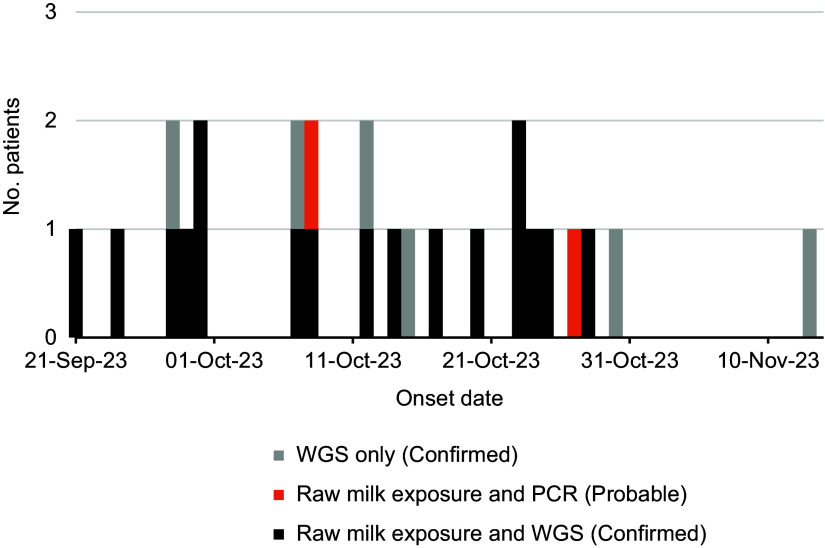
Epidemiologic curve of *Salmonella* cases by identification method and classification status (*N* = 25). Prepared by County of San Diego Health and Human Services Agency. Figure 3. long description.

**Figure 4.**
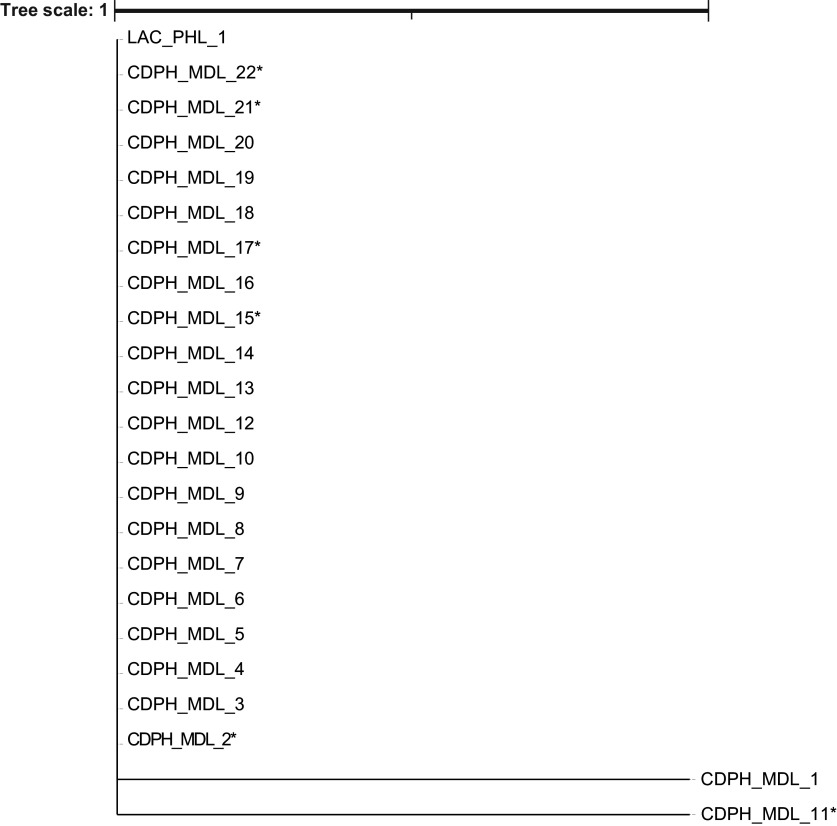
Dendrogram of core genome multilocus sequence typing (cgMLST) results for Salmonella isolates from San Diego County (*N* = 23) associated with unpasteurized dairy. The six Salmonella isolates indicated with an * were linked to this outbreak by WGS and did not report consumption of Farm A unpasteurized dairy products. The tree was generated by using BioNumerics version 7.6 and visualized using the interactive tree of life [13, 14]. Prepared by County of San Diego Health and Human Services Agency. Figure 4. long description.

By 31 January 2024, a total of 25 cases (23 confirmed and 2 probable cases) from San Diego County were identified with isolate dates from 23 September to 14 November 2023, with 19 (76%) reporting exposure to Farm A unpasteurized dairy products. Median patient age was 12 years (range: 1–47 years); 18 (72%) were male (Tables 1 and 2). All were CoSD residents with symptom onsets during 21 September–13 November 2023. A total of 15 (60%) had bloody diarrhea. Three patients (12%) were hospitalized; all three were children. Eighteen patients (72%) reported consumption of unpasteurized milk, three (12%) reported unpasteurized cheddar cheese, one (4%) reported unpasteurized kefir, and one (4%) reported unpasteurized heavy cream. Of confirmed cases, 17 reported consumption of Farm A unpasteurized dairy products, and six had *Salmonella* isolates linked to this outbreak by WGS and did not report consumption of Farm A unpasteurized dairy products (Figures 3 and 4). WGS results for the San Diego County culture-confirmed cases matched a cluster related to Farm A unpasteurized milk already under investigation by CDPH, involving 35 California local health jurisdictions and four other states. As of 15 October 2024, this larger cluster included 171 cases.

**Table 1.** Salmonellosis case characteristics (*N* = 25) – San Diego County, September 2023–January 2024 Table 1. long description.

Case characteristic	*n* (%)
Sex	
Male	18 (72%)
Female	7 (28%)
Hospitalized	3 (12%)
Deaths	0 (0%)
Symptoms*	
Diarrhea	23 (92%)
Stomach cramps	20 (80%)
Fever	17 (68%)
Bloody diarrhea	15 (60%)
Vomiting	12 (48%)
Exposure	
Farm A unpasteurized dairy	19 (76%)
Types of Farm A unpasteurized dairy*	
Milk	18 (72%)
Cheddar cheese	3 (12%)
Kefir	1 (4%)
Heavy cream	1 (4%)
Culture-confirmed	23 (92%)
PCR-positive	2 (8%)

PCR, polymerase chain reaction.

* Not mutually exclusive.

**Table 2.** WGS results for the 23 confirmed *Salmonella* isolates [12] Table 2. long description.

NCBI accession	SRR ID	WGS ID
SAMN37875612	SRR26424240	PNUSAS393203
SAMN38042472	SRR26588455	PNUSAS396758
SAMN38042471	SRR26588117	PNUSAS396764
SAMN38253135	SRR26825728	PNUSAS400881
SAMN38253134	SRR26826427	PNUSAS400882
SAMN38351632	SRR26906757	PNUSAS403121
SAMN38253131	SRR26826515	PNUSAS400885
SAMN38471447	SRR26969146	PNUSAS404210
SAMN38122322	SRR26684765	PNUSAS398870
SAMN38253167	SRR26826329	PNUSAS400901
SAMN38250490	SRR26825366	PNUSAS399314
SAMN38812608	SRR27200093	PNUSAS408336
SAMN38450628	SRR26958020	PNUSAS403826
SAMN38450629	SRR26958012	PNUSAS403827
SAMN38450627	SRR26958013	PNUSAS403828
SAMN38812597	SRR27200097	PNUSAS408347
SAMN38649350	SRR27050916	PNUSAS406033
SAMN38793092	SRR27190792	PNUSAS407762
SAMN38649355	SRR27050935	PNUSAS406042
SAMN38649367	SRR27050944	PNUSAS406029
SAMN38815849	SRR27202970	PNUSAS408542
SAMN39125248	SRR27351715	PNUSAS410942
SAMN39472603	SRR27593251	PNUSAS414945

NCBI Accession, National Center for Biotechnology Information accession number; SRR ID, Sequence Read Archive run identification number; WGS ID, whole-genome sequencing identification number.

### Environmental public health actions

One week after the start of the outbreak investigation, Farm A’s raw milk and raw heavy cream were recalled. CoSD issued an updated media release to San Diego County residents sharing the recall information and best-by dates 11 October–6 November 2023 and directing readers to CDPH’s online California Food Recall Information Sheet for specific product identification numbers and the retailer distribution list. The media release also included the updated count of infected patients and restated the risks for unpasteurized dairy product consumption [15]. Retailers were notified that nonrecalled Farm A products could be released from the pull-and-hold request. Production resumed on 31 October 2023.

### Environmental results

The sample of implicated product collected from one infected patient in COSD tested negative for *Salmonella.* Samples of unpasteurized dairy products, including unpasteurized milk, collected by CDFA from Farm A on 19 October and 25 October 2023, were found to be positive for *S. Typhimurium*, matching the outbreak strain by WGS. As part of the larger cluster investigation by CDPH, CDPH FDB collected environmental samples of Farm A’s unpasteurized dairy products from vendors. One of these samples of unpasteurized milk tested positive for *S. Typhimurium.*

## Discussion

In October 2023, CoSD identified this outbreak of salmonellosis among persons consuming Farm A’s unpasteurized dairy products. CoSD released early advisories warning of Farm A-associated risks, which might have prevented additional infections. These advisories recommended that individuals follow up with their healthcare providers if they became symptomatic. Throughout the outbreak response, strong collaboration between epidemiologic, environmental health, and laboratory partners enabled prompt partner notifications, timely communication with local Farm A unpasteurized dairy product vendors, and product collection and testing from retailers and an infected person. The prompt communication and collaborative response are evident by the limited time that elapsed from initiation of the outbreak investigation on 18 October to issuance of the producer’s voluntary recall on 24 October.

WGS results enabled further identification of cases who did not report consumption of Farm A unpasteurized dairy products. Identification of cases was particularly important during this outbreak investigation because of the multiple retailers distributing Farm A’s unpasteurized dairy products (Figure 3) throughout California. Additionally, WGS results from this outbreak investigation matched results from a cluster already under investigation by CDPH related to Farm A unpasteurized milk. Use of WGS methods allows for comparison of clusters of infection over time and across jurisdictions by indicating genetic relatedness. WGS can be used for creating sensitive and specific case definitions and for identifying linkages to an implicated source of infection [16].

Before this outbreak, CDFA had announced two food recalls in 2023 linked to unpasteurized dairy products produced by Farm A, a company located in Fresno County, California [17, 18]. The first recall occurred after the detection of *Campylobacter jejuni* and was for Farm A raw whole milk with the best-by date of 5 May 2023 [17]. The second recall was for Farm A raw cheddar cheese with the best-by date of 16 March 2024, which occurred after *Salmonella* detection and was announced on 1 August 2023 [18]. After the outbreak in 2024, Farm A issued a voluntary recall of raw cheddar cheese, lasting from 16 February 2024 to 23 February 2024, linked to a multistate *E. coli* O157:H7 outbreak [19]. In November 2024, CDPH shared voluntary recalls of two Farm A cream top, whole raw milk lots with best-by dates of 27 November 2024 and 7 December 2024, as a result of bird flu virus detection [20, 21]. When asked about prevention efforts against illnesses linked to unpasteurized dairy consumption, state health and agriculture department respondents indicated the need for further prevention resources and education materials [22].

Although CoSD responded promptly and used strong partner collaborations, infected San Diego County patients continued to be identified through January 2024. Illness onset dates ranged from 21 September 2023 to 13 November 2023. Based on the information available on 19 October, grocery vendors were asked to voluntarily pull and hold Farm A unpasteurized dairy products. After Farm A’s voluntary recall of raw milk and raw heavy cream on 24 October, retailers were updated that non-recalled products could be released from the pull-and-hold request. Production resumed 1 week after the recall. Infected patients may have continued to be identified after the voluntary recall due to potential gaps in risk communication or secondary patients.

In total, 25 patients from San Diego County were identified and linked to this *Salmonella* outbreak, with 76% reporting exposure to Farm A unpasteurized dairy products. Children are at an increased risk of *Salmonella* infection and severe illness. In this outbreak, the three patients requiring hospitalization were children. During this investigation, at-risk groups for severe illness were important to identify for prevention and focused messaging about the risk of consuming unpasteurized products.

Timely public advisories may be helpful when investigating a salmonellosis outbreak to prevent additional exposures after epidemiologic data have implicated a product associated with illness. Additionally, prevention and education with a focus on risk communication aimed at persons at an increased risk for severe illness can help minimize future outbreaks linked to unpasteurized dairy products. In California, educating consumers to look for the warning label included on all unpasteurized dairy products may increase awareness and alert them to the risk of harmful bacterial exposure.

## Data Availability

All serotype and WGS data have been submitted to NCBI. All NCBI Accession numbers, Sequence Read Archive run identification numbers (SRR ID), and whole-genome sequencing identification numbers (WGS ID) are listed in Table 2.

## Long descriptions

### Figure 1. Long description

A two-panel photo of a white plastic milk jug.

Left panel: The front of the jug. A white label features a red banner reading CREAM TOP - WHOLE. Below this, text states FROM GRASS-GRAZED COWS. A row of icons includes SOY free, Non-G M O Project Verified, Certified Humane, and Family Owned. At the bottom is a photo of a cow in a field and a circular seal that says TEST and HOLD.

Right panel: The back of the jug. The label is divided into several sections:

- Top section: HALF GALLON | 64 oz | WHOLE RAW MILK. INGREDIENTS: whole raw milk.

- Middle section: A black banner with white text about grass-grazing practices and an F D A disclaimer regarding r B S T.

- Nutrition Facts table: Lists Serving Size 1 cup (240 m L), 150 Calories, 8 grams Total Fat, 30 milligrams Cholesterol, 105 milligrams Sodium, 12 grams Total Carbohydrate, and 8 grams Protein.

- Lower section: Mentions 64 O Z NON-TOXIC PACKAGING (B P A, B P S, BISPHENOL, PHTHALATES FREE). A black box states RAW PROBIOTICS MAY HELP WITH DIGESTIVE HEALTH next to a Kosher symbol and a barcode (8 35204 00009 5).

- Bottom section: A bold WARNING regarding the risks of consuming raw unpasteurized milk for certain populations like children, the elderly, and pregnant women.

Navigate back to Figure 1..

### Table 1. Long description

The table consists of two columns: Case characteristic and n (%).

Demographics and Outcomes:

- Sex: 18 males (72%) and 7 females (28%).

- Hospitalized: 3 (12%).

- Deaths: 0 (0%).

Symptoms (not mutually exclusive):

- Diarrhea: 23 (92%).

- Stomach cramps: 20 (80%).

- Fever: 17 (68%).

- Bloody diarrhea: 15 (60%).

- Vomiting: 12 (48%).

Exposure Data:

- Farm A unpasteurized dairy: 19 (76%).

- Types of Farm A unpasteurized dairy (not mutually exclusive): Milk 18 (72%), Cheddar cheese 3 (12%), Kefir 1 (4%), and Heavy cream 1 (4%).

Diagnostic Confirmation:

- Culture-confirmed: 23 (92%).

- P C R-positive: 2 (8%).

Footnote: P C R stands for polymerase chain reaction.

Navigate back to Table 1..

### Table 2. Long description

The table consists of three columns: N C B I accession, S R R I D, and W G S I D. There are 23 rows of data:

* Row 1: SAMN37875612, SRR26424240, PNUSAS393203

* Row 2: SAMN38042472, SRR26588455, PNUSAS396758

* Row 3: SAMN38042471, SRR26588117, PNUSAS396764

* Row 4: SAMN38253135, SRR26825728, PNUSAS400881

* Row 5: SAMN38253134, SRR26826427, PNUSAS400882

* Row 6: SAMN38351632, SRR26906757, PNUSAS403121

* Row 7: SAMN38253131, SRR26826515, PNUSAS400885

* Row 8: SAMN38471447, SRR26969146, PNUSAS404210

* Row 9: SAMN38122322, SRR26684765, PNUSAS398870

* Row 10: SAMN38253167, SRR26826329, PNUSAS400901

* Row 11: SAMN38250490, SRR26825366, PNUSAS399314

* Row 12: SAMN38812608, SRR27200093, PNUSAS408336

* Row 13: SAMN38450628, SRR26958020, PNUSAS403826

* Row 14: SAMN38450629, SRR26958012, PNUSAS403827

* Row 15: SAMN38450627, SRR26958013, PNUSAS403828

* Row 16: SAMN38812597, SRR27200097, PNUSAS408347

* Row 17: SAMN38649350, SRR27050916, PNUSAS406033

* Row 18: SAMN38793092, SRR27190792, PNUSAS407762

* Row 19: SAMN38649355, SRR27050935, PNUSAS406042

* Row 20: SAMN38649367, SRR27050944, PNUSAS406029

* Row 21: SAMN38815849, SRR27202970, PNUSAS408542

* Row 22: SAMN39125248, SRR27351715, PNUSAS410942

* Row 23: SAMN39472603, SRR27593251, PNUSAS414945

Navigate back to Table 2..

### Figure 3. Long description

The y-axis is labeled No. patients with increments from 0 to 3. The x-axis is labeled Onset date with major ticks for 21-Sep-23, 01-Oct-23, 11-Oct-23, 21-Oct-23, 31-Oct-23, and 10-Nov-23.

The legend identifies three categories:

* Gray bars: W G S only Confirmed.

* Orange bars: Raw milk exposure and P C R Probable.

* Black bars: Raw milk exposure and W G S Confirmed.

Data distribution from left to right:

* Late September: Single black bars appear on Sep 21 and Sep 24. A cluster around Sep 28-30 shows a peak of 2 cases, including one gray and one black bar.

* Early October: A peak of 2 cases occurs on Oct 7 with one black and one orange bar. Another peak of 2 cases occurs on Oct 12 with one black and one gray bar.

* Mid to Late October: Sporadic single black bars occur between Oct 14 and Oct 21. A peak of 2 black bars occurs on Oct 23. A single orange bar appears on Oct 27.

* Late October to November: Single gray bars appear on Oct 30 and Nov 13.

The overall trend shows a sporadic distribution of cases with small clusters of 2 patients occurring roughly every 5 to 10 days through October.

Navigate back to Figure 3..

### Figure 4. Long description

A phylogenetic tree diagram with a scale bar at the top indicating a tree scale of 1. The diagram consists of a vertical line on the left from which horizontal branches extend to the right to label specific isolates.

Twenty-one isolates are clustered together at the top with zero branch length, indicating identical or near-identical c g M L S T profiles. These are listed from top to bottom as:

* L A C underscore P H L underscore 1

* C D P H underscore M D L underscore 22 asterisk

* C D P H underscore M D L underscore 21 asterisk

* C D P H underscore M D L underscore 20

* C D P H underscore M D L underscore 19

* C D P H underscore M D L underscore 18

* C D P H underscore M D L underscore 17 asterisk

* C D P H underscore M D L underscore 16

* C D P H underscore M D L underscore 15 asterisk

* C D P H underscore M D L underscore 14

* C D P H underscore M D L underscore 13

* C D P H underscore M D L underscore 12

* C D P H underscore M D L underscore 10

* C D P H underscore M D L underscore 9

* C D P H underscore M D L underscore 8

* C D P H underscore M D L underscore 7

* C D P H underscore M D L underscore 6

* C D P H underscore M D L underscore 5

* C D P H underscore M D L underscore 4

* C D P H underscore M D L underscore 3

* C D P H underscore M D L underscore 2 asterisk

At the bottom of the tree, two isolates are separated by long horizontal branches, indicating greater genetic distance from the main cluster. These are:

* C D P H underscore M D L underscore 1

* C D P H underscore M D L underscore 11 asterisk

Navigate back to Figure 4..
